# Psychiatric neuroimaging designs for individualised, cohort, and population studies

**DOI:** 10.1038/s41386-024-01918-y

**Published:** 2024-08-14

**Authors:** Martin Gell, Stephanie Noble, Timothy O. Laumann, Steven M. Nelson, Brenden Tervo-Clemmens

**Affiliations:** 1 Department of Psychiatry, Psychotherapy and Psychosomatics, Faculty of Medicine, RWTH Aachen University, Aachen, Germany; 2 Institute of Neuroscience and Medicine (INM-7: Brain & Behaviour), Research Centre Jülich, Jülich, Germany; 3 Masonic Institute for the Developing Brain, University of Minnesota, Minneapolis, MN USA; 4 Psychology Department, Northeastern University, Boston, MA USA; 5 Bioengineering Department, Northeastern University, Boston, MA USA; 6 Center for Cognitive and Brain Health, Northeastern University, Boston, MA USA; 7 Department of Psychiatry, Washington University School of Medicine, St. Louis, MO USA; 8 Department of Pediatrics, University of Minnesota, Minneapolis, MN USA; 9 Department of Psychiatry and Behavioral Sciences, University of Minnesota, Minneapolis, MN USA; 10 Institute for Translational Neuroscience, University of Minnesota, Minneapolis, MN USA

**Keywords:** Cognitive neuroscience, Human behaviour

## Abstract

Psychiatric neuroimaging faces challenges to rigour and reproducibility that prompt reconsideration of the relative strengths and limitations of study designs. Owing to high resource demands and varying inferential goals, current designs differentially emphasise sample size, measurement breadth, and longitudinal assessments. In this overview and perspective, we provide a guide to the current landscape of psychiatric neuroimaging study designs with respect to this balance of scientific goals and resource constraints. Through a heuristic data cube contrasting key design features, we discuss a resulting trade-off among small sample, precision longitudinal studies (e.g., individualised studies and cohorts) and large sample, minimally longitudinal, population studies. Precision studies support tests of within-person mechanisms, via intervention and tracking of longitudinal course. Population studies support tests of generalisation across multifaceted individual differences. A proposed reciprocal validation model (RVM) aims to recursively leverage these complementary designs in sequence to accumulate evidence, optimise relative strengths, and build towards improved long-term clinical utility.

## Introduction

The “traditional” model in psychiatric neuroimaging, characterised by small, cross-sectional, and observational studies has historically dominated research endeavours. Over the last decade, numerous challenges to this model have been presented, including reproducibility, inference, reliability and power [1–8]. These challenges have raised concern for the ultimate potential clinical utility of neuroimaging (particularly widely used non-invasive techniques like structural and functional MRI [fMRI]) to advance the aetiology or treatment of psychiatric disorders. As a result, there have been recent calls for a shift toward either population-level “big data” or more targeted precision studies, often longitudinal in nature [9–11]. Another perspective on this shift is to acknowledge that there is no “free lunch” in any study design, as practical (e.g., participant recruitment) and financial constraints (e.g., costs associated with neuroimaging studies) prevent researchers from achieving the largest possible participant numbers, longitudinal time points, and breadth of assessments [9, 10, 12]. While these are important considerations for neuroimaging, balancing ideal study designs and resource constraints likewise drive many other areas of psychiatric and medical research [13]. Investigators should carefully consider these trade-offs to achieve robust and reproducible psychiatric neuroimaging and ultimately increase potential clinical utility.

In this overview and perspective, we provide a guide to the current landscape of psychiatric neuroimaging study designs with respect to a balance of scientific goals and resource constraints. We outline methodological considerations among common designs, highlighting an emerging global trade-off in within-person precision and between-person generalisability. To conclude, we propose a reciprocal validation model (RVM) that aims to leverage small-sample precision studies and large-sample population studies in a recursive sequence towards evidence accumulation and long-term clinical utility.

## Study designs

**Fig. 1:**
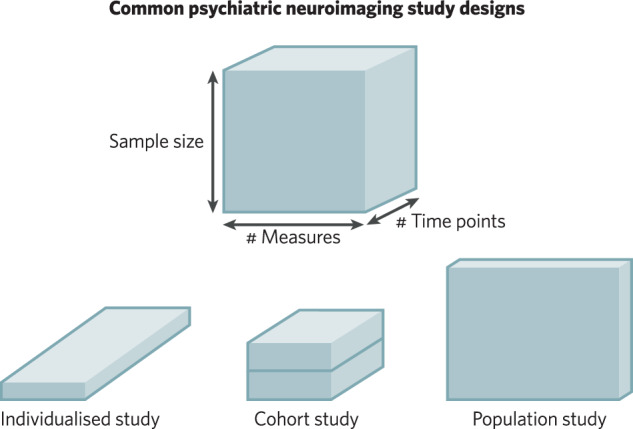
Common psychiatric neuroimaging designs via heuristic data cube. A balance of scientific goals and resource constraints leads common psychiatric neuroimaging studies to differentially emphasise design features. Prototypical examples of psychiatric neuroimaging designs are displayed according to the dimensions of sample size (y-axis), the number of different measures collected (x-axis), and the number of time points assessed (z-axis).

Common designs in psychiatric neuroimaging can be visualised using a heuristic data cube [14, 15] (Fig. 1). In this illustration, each dimension represents a design choice: study sample size, measurement breadth (e.g., cognitive tasks, self-report scales), and measurement time points (e.g., longitudinal assessments). Owing to the balance of scientific goals and resource constraints, intensive longitudinal studies for example tend to have smaller samples (e.g., single-participant, cohort studies), whereas large sample population studies often have very few, or just a single, assessment time point. Likewise, population studies typically focus on a broad set of potential measures or constructs, whereas targeted single-participant or cohort studies emphasise more precise estimates (i.e., higher reliability and convergent validity) of fewer variables or constructs. Thus, while any combination of design features is, in principle, possible, finite resources lead to prototypical examples that differentially emphasise study design features.

When examined in isolation or through the lens of a specific research question, relative strengths and weaknesses of a given study design may appear as a much-needed emphasis or a fundamental flaw. Recent perspectives have highlighted the importance of large sample sizes, underscoring the value of more inclusive samples and generalisability towards real-world complexity [16–18]. Others have recognised that investing in such samples may critically come at the cost of measurement precision and carefully targeted assessments [9, 19] and that even in recent large-scale population samples such inclusivity is not guaranteed [20]. Critically, common designs should support distinct but complementary research questions and inferential goals. While misalignment between research questions and study designs precipitates statistical issues and leads to inappropriate inferences, proper alignment is possible for all designs. Further, acknowledging the high resource demands of neuroimaging, leveraging the relative strengths and complementary insights from multiple designs is essential for improvements to reproducibility and evidence building. To illustrate these points, we consider prototypical examples from three psychiatric neuroimaging designs: single-participant or individualised studies, cohort or group studies, and population-level “big data” studies. These are not meant to exhaustively capture the current state of the literature, but rather serve as an overview of common designs.

The focus here is on psychiatric neuroimaging, operationalised as studies examining neural markers of treatment, longitudinal course, or correlates of disorders/symptoms. Where relevant, studies of normative brain variability and development are also highlighted as they provide context for clinical presentations and broader consideration of brain-behaviour methods. Further, this work emphasises study design and corresponding methodological and inferential trade-offs, though it necessarily discusses related issues of measurement reliability, validity, and prediction. We discuss these in context and refer the reader to existing literature [18–25].

## Single-participant and individualised studies

Single-participant and individualised designs analyse a single participant or a series of individuals over repeated sessions, and often multiple contexts [21, 22]. In this way, they prototypically aim to maximise the time point dimension (z-axis) of the theoretical data cube (Fig. 1—left). This design is akin to a case study in psychiatry or medicine that describes clinical phenomena and their longitudinal course. Key to individualised designs is that the unit of analysis is a single participant that often “serves as their own control” across time, contexts or experimental manipulations. This importantly differs from longitudinal cohorts (see below) where inferences are made at the group level or “on average”.

The major advantages of this design are a potentially high degree of experimental control and flexibility, as measurements and manipulations can be precisely tailored to the individual or phenomena under study. This makes individualised studies potentially amenable to both hard-to-recruit patient populations as well as more complex study protocols. To this end, an emphasis on intensive longitudinal assessments well aligns this design with deep behavioural phenotyping or precision functional neuroimaging approaches that collect large amounts of data within an individual to maximise measurement reliability (i.e., the relative stability or consistency of assessments) [22–24] (see also [26]). Together, these strengths make individualised studies particularly well suited for investigating within-person mechanistic insights by establishing interventional and longitudinal links between neuroimaging metrics and symptom course. An investigator interested in brain changes relevant to depression treatment, for example, might use an individualised design with precision neuroimaging before, during, and after treatment using transcranial magnetic stimulation (TMS) to investigate person-specific functional connectivity changes. This is in contrast to a cohort study, where inferences might be drawn about  average effects among a group of patients receiving TMS (note that group effects do not necessarily translate to individual-level findings - see [8] and below sections for details), or a population study that might examine variation among typical treatments in standard care (i.e., not determined by the study protocol).

**Fig. 2:**
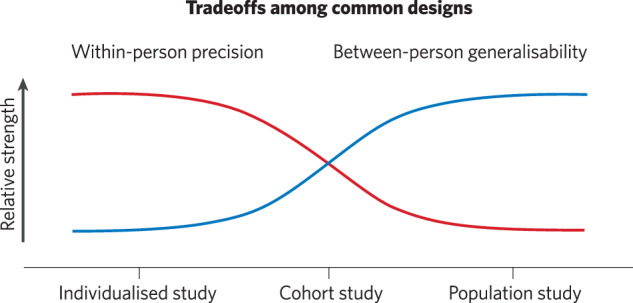
Trade-offs among common psychiatric neuroimaging designs. Due to finite resources and varying scientific goals, prototypical examples of psychiatric neuroimaging designs differentially emphasise within-person precision (e.g., measurement reliability, internal validity, and the potential for experimental control) compared to between-person generalisability (e.g., the potential for a sample to capture “real-world complexity”). Note the y-axis denotes a heuristic relative scale, where lower ‘values’ convey relatively lower (not an absolute absence of) within-person precision or between-person generalisability.

While individualised studies offer the upside of drawing longitudinal inferences on the level of an individual participant, methodological challenges such as low statistical power for inferential statistics, and difficulties with interpreting single-subject effect sizes [21], often mean that generalisation to a broader population needs to be considered. That is, an emphasis on the time point dimension often comes at the cost of a larger sample size that is required to determine whether the results would vary among other patients with the same presentation or as a function of salient sociodemographic or psychiatric factors (Fig. 2). Not all individualised studies will aim for such inferences at the outset (e.g., functional mapping of a specific patient for surgery), but subsequent clinical translation will require demonstrations of generalisability for the developed approach (e.g., person specific TMS targeting; forecasting model of symptom severity) or mechanism (e.g., longitudinal covariation between brain function and symptom course).

Recent empirical examples illustrating the utility of individualised studies in neuroimaging have followed a single individual weekly for 18 months to demonstrate changes in connectivity [25, 27] or daily to study functional changes throughout the menstrual cycle [28]. Most relevant to psychiatry, individualised precision neuroimaging studies have shown that brain network organisation varies significantly between individuals [22], and suggests plasticity even after large lesions early in life [29]. These reports also illustrate a more general claim that holds irrespective of a particular study design: large amounts of functional neuroimaging data are needed to reliably estimate individual brain topography that gets otherwise obscured when averaging across individuals [8, 22]. Ongoing work using multi-echo acquisition suggests opportunities to shorten the requisite time for delineating patient-specific functional neuroanatomy, although still utilises acquisitions longer than what may be typically acquired [30].

When precision individualised designs are paired with an experimental manipulation, as opposed to an observational design, they can offer within-person mechanistic insights. For example, a recent study [31] examined functional neuroimaging alterations during and after immobilisation of the upper extremity in three participants. These findings suggest that spontaneous activity pulses that emerged during arm immobilisation may contribute to maintaining connectivity within disused motor regions. Within psychiatry, recent investigations using densely sampled participants have indicated the impact of inter-subject variability in functional organisation on the effectiveness of transcranial magnetic stimulation (TMS); illustrating that coil placement guided by group average maps will stimulate different functional networks across subjects [32].

## Cohort and group studies

Cohort studies (also referred to as group designs) are arguably the predominant psychiatric neuroimaging design (Fig. 1—middle). In contrast to individualised designs, cohort studies make inferences about averages within a well-defined group (e.g., participants with a specific mental health presentation). Cohort studies therefore necessarily have a broader “sampling frame” (i.e., population drawn from to create the study sample: e.g., patients treated at a local clinic) and “target population” (i.e., a larger group that inferences derived from the study ought to generalise to: e.g., young adults with depression) [33, 34] than individualised studies. Typical sample sizes for this design range from tens to hundreds of participants (cf., openneuro.org) which is partly related to the considerable variety of potential cohort designs, including longitudinal cohorts of a single diagnostic group, case-control studies, and those comparing treatments within and/or between individuals [35]. For the current overview and guide to common trade-offs across psychiatric neuroimaging designs, we emphasise a prototypical case that can be viewed as moderately large and moderately longitudinal, relative to other designs (individualised and population studies; see Fig. 1).

Many traditional statistical approaches were developed with group comparisons in mind and are more powered in a cohort study compared to an individualised study when matched for longitudinal assessments and precision [21]. However, due to resource constraints, cohort studies and more standard neuroimaging designs have not historically achieved the same measurement precision and longitudinal time points [36]. Finally, while cohort studies offer higher generalisability compared to single-participant studies, they lack the very large sample sizes and real-world sociodemographic or psychiatric complexity targeted in large population studies (see below).

A major advantage of cohort studies for psychiatric neuroimaging lies in a potential balance of relatively high within-person precision (e.g., measurement reliability, internal validity, and potential for experimental control) while retaining some degree of between-person generalisability beyond a single-participant design (Fig. 2). Cohort studies can thus be well-suited to leverage this balance to characterise varied clinically relevant processes with careful longitudinal assessments, sample selection, and experimental conditions. Similarly to individualised designs, cohort studies can investigate within-person mechanisms by means of experimental manipulations (see also for detailed discussion [37–40]). Longitudinal and/or intervention-based designs (e.g., lesion mapping, TMS, pharmacological interventions or psychosocial treatments) are particularly well suited for this, as they can determine temporal precedence and track common clinical or biological change. Following the example above, an investigator interested in neural circuits of depression treatment might employ a cohort design with longitudinal neuroimaging to compare treatment and control groups before and after a TMS intervention. Critically, such a study should aim to balance within-person precision and overall sample size.

Cohort designs have been widely used, with varying success, to investigate circuit-level “mechanisms of action” underlying psychiatric treatments such as TMS [41], deep brain stimulation (DBS; [42, 43]) and pharmacological intervention [44]. Cohort designs have likewise been invaluable for characterising average changes in normative brain development during the lifespan period of adolescence when psychiatric disorders first emerge [45–49]. For example, structural neuroimaging in longitudinal developmental cohorts has demonstrated and replicated changes in multiple properties (grey and white matter volume, cortical thickness) by leveraging key design strengths in longitudinal data: relatively high reliability of structural neuroimaging measures (compared to other neuroimaging measures [50]) and large magnitude changes (see [51]). However, we note that to concurrently estimate both average developmental change and variability in development, as in normative modelling approaches, very large population samples are required [52–54]. Cohort studies can also be well suited to examine very focused questions among patient groups. For example, owing to the relatively low prevalence rate of many psychiatric disorders within a general population [55], a targeted cohort study, if appropriately powered, may be better equipped to assess heterogeneity within a given diagnostic group, or trajectories over time compared to population-based samples. Especially when paired with high precision, this can allow for individualised insights into underlying neural patterns [56]. Taken together, strengths of existing psychiatric neuroimaging cohort studies most often arise from leveraging longitudinal data with high-precision measurements to track common clinically relevant change (e.g., treatment) or biological change (e.g., development, ageing) [9, 10]. We note, however, that collecting such precision imaging and phenotypic data in clinical cohorts is currently rare [57].

Cohort studies have been significantly less successful at providing reproducible and robust associations between individual differences in measures of brain function and structure and mental health (e.g., associations between functional connectivity and depression symptom severity), and individual differences more generally. This is likely driven by the underlying effect sizes of brain-behaviour associations across the population, which have been shown to be relatively small, compared to traditional effect size thresholds [3, 58, 59]. Moreover, given the inherent complexity of both human behaviour and common non-invasive neuroimaging techniques (e.g., MRI/fMRI), such small effects are not unreasonable. As a result, these challenges in cohort studies may be viewed as a misalignment between the study design and the question of multifaceted individual differences that likely require high sample complexity and generalisability (see below). Even if clearly labelled as exploratory, correlation or prediction analyses in traditional single-site cohort studies with tens of participants are likely to be underpowered and consequentially produce false or inflated positive findings by chance. Cross-sectional individual differences should, therefore, nearly always be avoided in such studies and instead, investigated in a larger dataset using appropriate methods [60]. Nevertheless, the appropriateness of individual difference analyses within cohort studies will depend on the range of inter-individual variability in the behavioural and brain metrics captured in the cohort study. Therefore, investigators must consider the study sampling frame and whether results are expected to generalise beyond the specific sample or clinical group to the broader population when choosing a study design and interpreting brain-behaviour relationships.

## Population studies

Described as the intersection of neuroscience and epidemiology, population (or population-based, or sometimes referred to as “big data”) neuroimaging studies have the broadest sampling frame among designs, including a wide range of individuals with various psychiatric presentations (and/or risk factors) and sociodemographic backgrounds [16, 17]. Ideally, population studies accurately represent a complex target population (e.g., nationally representative of adolescents in the United States) through inclusive and well-planned sampling (cf., [34, 61]). Population studies thus emphasise sample size (typically thousands of participants or more) and measurement breadth (X & Y-axes of the heuristic data cube, Fig. 1 - right) and can have higher sample diversity and generalisability (see also [62]). Owing to the recurring theme of balancing scientific goals with resource constraints, however, this often comes at the expense of the number of longitudinal time points and measurement precision relative to other designs (Fig. 2). Likewise, while population studies can, in theory, be interventional (e.g., phase 3 clinical trial in medicine), in neuroimaging they have most often been observational.

Large and relatively more complex samples make population studies particularly well-suited to study multifaceted psychiatric, sociodemographic, and neuroimaging inter-individual differences. In contrast, the typical use of observational designs with few longitudinal assessments renders these studies less suitable for studying within-person mechanisms of longitudinal symptom course or treatment. As a result, population designs in psychiatric neuroimaging often emphasise observational, and frequently cross-sectional, correlations with psychiatric phenotypes or behavioural traits. An investigator might use a population sample to develop a neuroimaging-based diagnostic biomarker for depression, leveraging the larger and more inclusive (relative to other designs) sample to accurately integrate and validate multiple behavioural and neuroimaging risk factors.

The use of population samples towards the potential development of diagnostic biomarkers and cross-sectional prediction of behavioural traits has rapidly increased with the relatively recent arrival of multiple large-scale open neuroimaging datasets (for an overview see: [63, 64]). While most of these efforts are new and several studies have shown critical challenges with leveraging population neuroimaging in psychiatry, some approaches have shown early promise. For instance, results from an international biomarker challenge indicate that patients with Autism Spectrum Disorder can be reliably classified using structural and functional imaging with accuracy many fold larger than with genotyping [65]. Outside of diagnostic biomarker discovery, prediction approaches can be utilised to uncover robust multivariate associations between neuroimaging and behaviour [18, 66, 67]. Data aggregation efforts integrating population samples, as well as independent investigator-initiated studies, have also significantly advanced neural models of multiple psychiatric diagnoses (e.g., [68, 69]). The relatively broad and more inclusive sampling in population studies further aligns well with a focus on continuous transdiagnostic dimensions that span normative to clinical ranges [70, 71]. Perhaps most notably, large population samples have provided unparalleled resources towards methods development and opportunities for replication and reproducibility studies (e.g., [3, 18, 51, 66, 72–79]).

Despite the strengths of population studies, they are, of course, not without limitations. Favouring measurement breadth and sample size for several phenomena (e.g., all mental health disorders) often leads to a relative loss of within-participant measurement precision and depth for specific phenomena (e.g., carefully curated convergent indicators of depression). This raises concerns for measurement reliability and validity as well as efforts to directly apply inferences from population studies to more targeted, individualised or cohort designs. For example, observable population-based effects (e.g., from studies sampling individuals without consideration for a given psychiatric trait) may be smaller than those found with pre-defined “extreme group” comparisons where participants are selected based on pre-screening [80]. Thus, effects from standard population studies, with characteristically broad sampling frames (i.e., independent of a specific psychiatric symptom) that span normative to clinical ranges, may not directly generalise to patient cohort studies. However, this raises questions of inclusivity for the broader population outside of extreme groups, and whether relatively increased effects in extreme groups should be seen as an initial building block for evidence accumulation [80, 81] or as a potential artefact of dichotomising dimensions (see [82, 83]). A further challenge for population studies is that confound control at the level of study design, which is typical in cohort studies (e.g., by matching control groups), is not feasible in large observational studies that, instead, require careful post hoc confound control [84]. Similarly, heterogeneity within diagnostic categories [85] likely becomes more evident in large datasets with population sampling, as participant selection is intentionally broad [16, 17]. Finally, a focus on individual differences in population studies may magnify more general challenges to symptom measurement in psychiatry (cf., [86, 87]), as current population studies generally incorporate existing scales that often lack validation for such population sampling. Additionally, many cognitive and behavioural measures have been optimised for minimal individual differences and maximal group or context (e.g., task contrast) differences, which compromise reliability (e.g., [88]). As a consequence, such assessments will not be ideal for assessing inter-individual differences in brain function and behaviour due to attenuation by reliability, even with large population samples [89, 90].

## A reciprocal validation model for building evidence across complementary designs

**Fig. 3:**
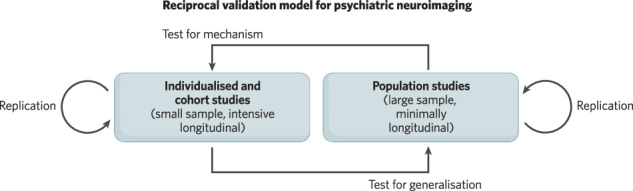
Reciprocal validation model for psychiatric neuroimaging. Common psychiatric neuroimaging designs have inherent methodological trade-offs (due to practical resource constraints and varying scientific goals) that can, nevertheless, be recursively sequenced to leverage relative strengths. A given psychiatric neuroimaging result may first emerge from a small sample, intensive longitudinal individualised or cohort study (e.g., a given brain region “A” changes with depression treatment) and be independently replicated with the same or similar design (left). The reciprocal validation model (RVM) emphasises the sequential testing of a conceptually related result (e.g., the link between depression symptoms and brain region “A”) to be tested for generalisability across individuals with a population study. Conversely, a psychiatric neuroimaging observation may start as an inter-individual difference neural correlate developed in a population sample (e.g., whole-brain connectivity correlate of depression) and be independently replicated with the same or similar population design. RVM emphasises testing this neural correlate for within-person “mechanisms” via interventions and tracking of precise longitudinal courses with individualised and cohort studies. We note that individualised and cohort studies are grouped in this figure based on the proposed shared study goal of within-person mechanisms. We refer the reader to earlier sections for further distinctions among these designs.

Even when carefully considering the relative strengths and weaknesses of individualised, cohort, and population study designs, clarity surrounding the decision to prioritise one over another can be elusive. Furthermore, comparing inferences across designs can be challenging, as group or population-level associations do not necessarily translate to individual-level mechanisms and changes observed at the individual level may not correspond to (or may even go in opposite directions as) those at the level of the whole sample (cf. ecological fallacy [91]). As a field, psychiatric neuroimaging currently lacks codified stages of evidence accumulation that shape such design choices and inferential goals in other resource-demanding areas. Clinical trials in broader medicine, as well as biomarker development in cancer research, for example, follow formal steps (cf., nih.gov; [92]), where new study designs, analogous to the reviewed psychiatric neuroimaging designs, are sequentially required for accumulating complementary evidence with prior success. Conversely, in less resource-demanding research areas (e.g., behavioural sciences) hybrid study designs, emphasising multiple axes of the data cube, and multiple simultaneous inferences, including those requiring multiple designs are more feasible. In this final section, we propose a reciprocal validation model (RVM) for how common psychiatric neuroimaging designs may be sequenced to accumulate evidence, leverage relative strengths, and build towards improved long-term clinical utility (Fig. 3).

An RVM sequence can start with observations from individualised and cohort studies that often prioritise intensive longitudinal data from relatively small sample sizes. While there are key distinctions among these designs (see preceding sections), both can provide within-person mechanistic insights that are essential for evidence building. For example, individualised and cohort designs that acquire a large amount of neuroimaging acquisition time and/or time points may identify novel longitudinal brain-behaviour associations (e.g., neural changes in brain region “A” track depression treatment). However, the extent to which the observed results generalise to large more complex samples may remain unclear. Therefore, subsequently testing aspects of this observation (e.g., the link between brain region “A” and depression symptoms) in a new or existing large, population study (in parallel and/or in addition to replication with a similar design) can clarify generalisability across key participant-level factors (e.g., attenuation of the association by parental income). This “test of generalisation” might then mirror stages of evidence accumulation in other related fields, such as in clinical trial development - where stage 3 identifies an effect in a well-controlled cohort study of hundreds, while stage 4 tests the effect in a large population sample of thousands. Recent work in broader neuroimaging has demonstrated the utility of this approach, with observations from targeted individual and cohort studies being tested for replication and generalisation in large population datasets [93]. Similarly, existing big datasets may be utilised as a bridge between group and population designs, as they can allow for subgroup analyses of a specific psychiatric condition as well as population-level individual differences.

An RVM sequence can also start with population studies that often prioritise large sample sizes at the cost of (relative to other designs) minimally longitudinal designs. For example, population samples may identify a neuroimaging feature or (more likely) a set of features that relate to individual differences in psychiatric symptoms in a cross-sectional sample (e.g., whole-brain multi-network connectivity with depression). Emphasising the large number of participants, these observations may have relatively high generalisability across participant-level features at the cost of within-person precision (measurement reliability and potential experimental control) and clearer evidence towards causality (temporal precedence and modifiability). Working to subsequently test findings from population studies in intensive longitudinal, and potentially interventional, individualised and cohort designs, offers a complementary inference towards temporal precedence and modifiability of the neural correlate. For example, an investigator may examine whether a neuroimaging spatial pattern predictive of depression symptoms, derived from a population study, changes in the context of depression treatment in a targeted cohort. Following a recursive sequence, this process could continue with further reciprocal testing between designs.

Recent concerns for rigour and reproducibility in psychiatric neuroimaging, underscore a goal to move from relatively early stages of evidence accumulation, “exploration”, to more advanced stages of “validation” (Fig. 3) or “confirmation” (see also [94, 95] for discussion). Use of the same general design with a new sample or new measures represents clear examples of such validation (“replication”; cf., [96] for a longer discussion on this terminology), as does assessing associations out-of-sample and, even more importantly, out-of-dataset in clinical prediction studies. Importantly, evidence accumulation in psychiatric neuroimaging will critically benefit from reciprocal validation and leveraging complementary designs in a recursive sequence. While reciprocal validation may not be feasible or warranted for all results (e.g., those with marginal significance or low replicability across studies within a given design [see Fig. 3]), an established framework via RVM can provide clarity on the current relative evidence accumulation.

Taken together, complementary designs with reciprocal validation can guide evidence accumulation towards long-term clinical utility. Resource constraints and varying inferential goals prevent a single study design or research team from simultaneously emphasising all axes of the data cube and advancing neuroimaging clinical utility in all manners of intervention, individualised tracking of longitudinal symptom course, or population-level clinical prediction. Nevertheless, appropriate alignment of such translational goals to specific study designs, each with inherent methodological trade-offs, and subsequent reciprocal validation with alternate approaches can provide a clearer path forward. While the long-term clinical utility of psychiatric neuroimaging remains unknown, a formalised evidence accumulation framework, like the proposed reciprocal validation model, is essential to organise these efforts and quantify progress.

## Conclusion

Psychiatric neuroimaging faces challenges in rigour and reproducibility that prompt reconsideration of the relative strengths and limitations of current designs. As reviewed through a heuristic data cube, a balance of scientific goals and resource constraints leads common psychiatric neuroimaging designs to differentially emphasise sample size, the number of measures or constructs, and the number of time points assessed. Investigators must be familiar with such trade-offs to ensure an appropriate alignment between research questions, designs and analyses. We emphasise a resulting global trade-off among common designs in within-person precision (relatively high in individualised, moderate in cohort studies, low in population studies) and between-person generalisability (relatively high in population studies, moderate in cohort studies, low in individualised studies). A proposed reciprocal validation model (RVM) aims to recursively leverage complementary designs in sequence to accumulate evidence, optimise relative strengths, and build towards improved long-term clinical utility.
